# Construction of differential mRNA-lncRNA crosstalk networks based on ceRNA hypothesis uncover key roles of lncRNAs implicated in esophageal squamous cell carcinoma

**DOI:** 10.18632/oncotarget.13828

**Published:** 2016-12-09

**Authors:** Shuang Yang, Qianqian Ning, Guobin Zhang, Hong Sun, Zhen Wang, Yixue Li

**Affiliations:** ^1^ Department of Bioinformatics and Biostatistics, School of Life Sciences and Biotechnology, Shanghai Jiao Tong University, Shanghai, China; ^2^ Key Lab of Computational Biology, CAS-MPG Partner Institute for Computational Biology, Shanghai Institutes for Biological Sciences, Chinese Academy of Sciences, Shanghai, China; ^3^ Shanghai Center for Bioinformation Technology, Shanghai, China; ^4^ Biomedical Information Research Center, Children’s Hospital of Shanghai; ^5^ Collaborative Innovation Center for Genetics and Development, Fudan University, Shanghai, China

**Keywords:** intrinsic subtype, competing endogenous RNA network, biomarker, lncRNA, ESCC, Pathology Section

## Abstract

Increasing evidence has indicated that lncRNAs acting as competing endogenous RNAs (ceRNAs) play crucial roles in tumorigenesis, metastasis and diagnosis of cancer. However, the function of lncRNAs as ceRNAs involved in esophageal squamous cell carcinoma (ESCC) is still largely unknown. In this study, clinical implications of two intrinsic subtypes of ESCC were identified based on expression profiles of lncRNA and mRNA. ESCC subtype-specific differential co-expression networks between mRNAs and lncRNAs were constructed to reveal dynamic changes of their crosstalks mediated by miRNAs during tumorigenesis. Several well-known cancer-associated lncRNAs as the hubs of the two networks were firstly proposed in ESCC. Based on the ceRNA mechanism, we illustrated that the“loss” of *miR-186-*mediated *PVT1*-mRNA and *miR-26b*-mediated *LINC00240*-mRNA crosstalks were related to the two ESCC subtypes respectively. In addition, crosstalks between *LINC00152 and EGFR*, *LINC00240 and LOX* gene family were identified, which were associated with the function of “response to wounding” and “extracellular matrix-receptor interaction”. Furthermore, functional cooperation of multiple lncRNAs was discovered in the two differential mRNA-lncRNA crosstalk networks. These together systematically uncovered the roles of lncRNAs as ceRNAs implicated in ESCC.

## INTRODUCTION

In the past decade, research on the non-coding RNA has gained widespread attention. lncRNAs are a large class of non-coding RNAs, which are longer than 200 nucleotides and pervasively transcribed in the genome. Currently, about 15,767 lncRNAs have been annotated in the human genome (GENCODE 24). lncRNAs play important roles in chromatin remodeling, transcriptional repression and post-transcriptional regulation [1]. Dysregulation of lncRNAs are associated with different types of cancers, and lncRNAs as reliable biomarkers for cancers are also identified [2–4]. However, due to functional diversity of lncRNAs, identification of cancer-related lncRNAs still faces challenges [5].

Esophageal squamous cell carcinoma (ESCC) as one of main forms of esophageal cancer is a highly aggressive solid tumor with poor prognosis and widely occurs in Asian countries [6]. Despite advances in the various diagnosis and treatment, the 5-year survival rate of ESCC remains only approximately 30% for patients after surgery [7]. Recent whole-genome and whole-exome sequencing on ESCC patients revealed six well-known tumor-associated genes (*TP53*, *CDKN2A* etc.) and two novel oncogenes (*ADAM29* and *FAM135B*) [8]. In non-coding RNA research, miRNAs (*miR-20 and miR-21* etc.) and lncRNAs (*HOTAIR* and *H19* etc.) as oncogenes or tumor suppressors were also studied in the development of ESCC [9–11]. In addition, using computational methods to predict lncRNA functions in ESCC has also been reported [12]. Even so, compared to coding genes and miRNAs, dysregulated lncRNAs implicated in ESCC remain little known.

Recently, the concept of competing endogenous RNAs (ceRNAs) indicates that RNA molecules harboring miRNA response elements (MREs) can communicate with each other by competing for common miRNA [13]. Especially, large-scale cross-linking immunoprecipitation (CLIP) experiments have identified thousands of miRNA-lncRNA interactions, which imply that miRNA-mediated crosstalks between mRNAs and lncRNAs widely exist in various biological processes [14]. Therefore, ceRNA hypothesis provides a new perspective to account for the function of as yet uncharacterized lncRNAs involved in ESCC.

A recent study discussed three scenarios of identifying cancer-related lncRNAs based on ceRNA network, including the “loss” and “gain” of miRNA-mediated mRNA-lncRNA crosstalks, and no significant change in mRNA-lncRNA crosstalks but their expression levels change in opposite directions [15]. However, most studies were related with the third class of dysregulation [16–19], and dynamic changes of the crosstalks between mRNAs and lncRNAs during tumorigenesis have never been studied. In addition, cancer subtype-specific ceRNA network was rarely considered before. In the present study, mRNA and lncRNA expression patterns of ESCC were identified, laying a foundation for discovering subtype-specific mRNA-lncRNA crosstalks. Furthermore, ESCC subtype-specific differential mRNA-lncRNA crosstalk networks mediated by miRNAs were constructed to compare lncRNA-related ceRNA networks in ESCC tissues and adjacent normal tissues. Our study uncovered ESCC-associated lncRNAs and shed light on mRNA-lncRNA crosstalks involved in tumorigenesis of ESCC.

## RESULTS

### Characteristics of subtypes of ESCC

**Figure 1:**
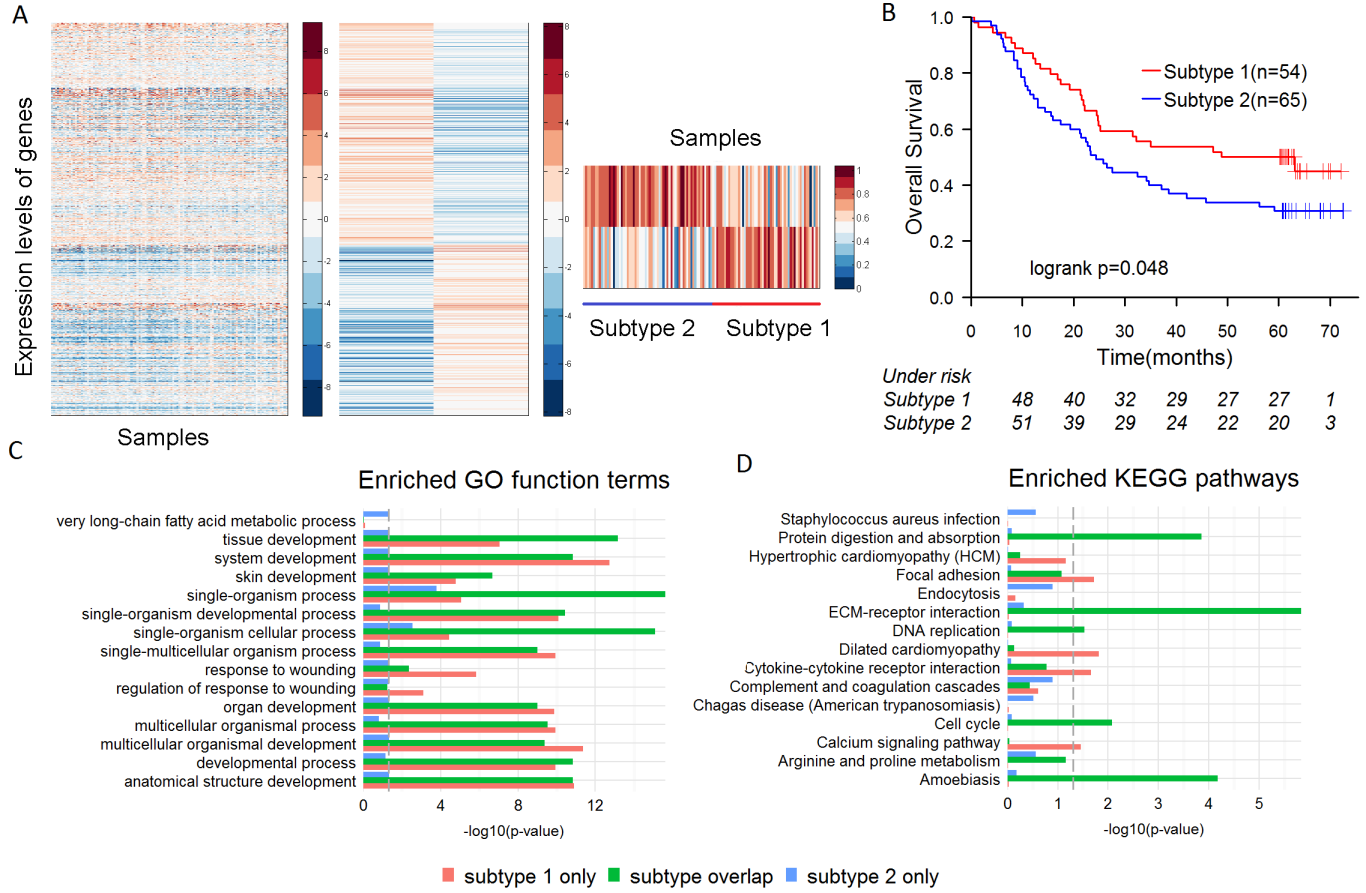
The subtypes of ESCC and their characteristics **A**. semi-NMF clustering identifies two subtypes of ESCC based on expression levels of lncRNAs and mRNAs. **B**. Overall survival of different subtypes of ESCC by Kaplan-Meier plot. ESCC subtypes are highly correlated with overall survival outcomes. **C**.-**D**. Differential expressed genes (DEGs) in two subtypes were divided into three groups, including the DEGs only in the subtype 1, the DEGs only in the subtype 2 and the DEGs in both subtypes. Significantly enriched GO terms and KEGG pathways of each group are shown.

We performed an unsupervised hierarchical clustering analysis based on the expression of mRNAs and lncRNAs of 119 ESCC patients. Semi-NMF analysis identified 2 intrinsic subtypes of ESCC, which contained 54 and 65 samples respectively (Figure 1A). Analysis of the survival prognosis after surgery showed that the overall survival time of patients in the subtype 1 was significantly longer than patients in the subtype 2 (median survival 41.4 *vs*. 33.5 months, *p* = 0.048) (Figure 1B). Fisher's exact test revealed the subtypes are significantly correlated with tumor grade (*p* = 3.55e-05), but not with other clinical features (Table S1). Patients with poorly differentiated ESCC were more prevalent in the subtype 2 than 1 (43.1% *vs*. 7.4%) (Table S2). In addition, cox regression analysis indicated that the subtypes of ESCC (HR = 1.601, 95% CI 0.998 to 2.569, *p* = 0.048) were more significantly correlated with overall survival of the patients than tumor grade (HR = 1.162, 95% CI 0.6264 to 2.157, *p* = 0.222).

**Table 1:** The top 10 genes in betweenness, degree, closeness and *DCLs* enrichment of differential mRNA-lncRNA crosstalk networks

	Betweenness	Degree	Closeness	DCLs enrichment	Overlaps
Network 1	***PVT1***	***PVT1***	***PVT1***	***PVT1***	***PVT1***
	*SNHG14*	*SNHG14*	*SNHG14*	***LINC00152***	*SNHG14*
	*RP11-834C11.4*	*RP11-834C11.4*	*SLC16A14*	*LINC00689*	*RP11-834C11.4*
	*LINC00240*	*LINC00689*	*RP11-834C11.4*	*RP11-834C11.4*	*LINC00240*
	***LINC00152***	***LINC00152***	*LINC00240*	*SNHG14*	***LINC00152***
	*BOLA3-AS1*	***FENDRR***	***LINC00152***	***FENDRR***	***FENDRR***
	*LINC00689*	*LINC00240*	*PPFIA1*	*BOLA3-AS1*	*BOLA3-AS1*
	***FENDRR***	*BOLA3-AS1*	***FENDRR***	***HOXA-AS2***	
	***HOXA-AS2***	***HOXA-AS2***	*BOLA3-AS1*	H19	
	*EPB41L4A-AS1*	*MAGI2-AS3*	*IKBIP*	*LINC00240*	
Network 2	*LINC00240*	*LINC00240*	*LINC00240*	*LINC00240*	*LINC00240*
	***SNHG1***	***SNHG1***	*SEMA6D*	*AP000525.9*	***SNHG1***
	*RP11-361F15.2*	*AP000525.9*	***SNHG1***	*AC156455.1*	*RP11-361F15.2*
	*AP000525.9*	*RP11-588K22.2*	*RP11-588K22.2*	*RP11-588K22.2*	*AP000525.9*
	*RP11-588K22.2*	*RP11-361F15.2*	*PCDH19*	*RP11-677M14.3*	*RP11-588K22.2*
	*RP11-677M14.3*	*AC156455.1*	*AP000525.9*	*RP11-361F15.2*	*AC156455.1*
	*SNHG14*	*SNHG14*	*PTPDC1*	***SNHG1***	
	*AC156455.1*	*AC009948.5*	*AC156455.1*	*FLG-AS1*	
	*FLG-AS1*	***FENDRR***	*RP11-361F15.2*	*RP11-115C21.2*	
	*AC009948.5*	*RP11-677M14.3*	*MET*	*TINCR*	

Overlaps contains the lncRNAs that appeared in each dimension. Cancer-related lncRNAs were highlighted in bold font.

**Table 2:** Correlations between any two lncRNAs within the modules in the different cell types

	Pairs	Adjacent tissues		Tumor tissues	
		CC	q-value	CC	q-value
Module 1	BOLA3-AS1-TRAF3IP2-AS1	0.32	0.016	-0.27	0.15
	TRAF3IP2-AS1-LINC00689	0.66	1.81e-07	0.23	0.18
	LINC00689-BOLA3-AS1	0.37	0.011	-0.02	0.87
Module 2	LINC00240-AP000525.9	0.61	3.74e-07	0.28	0.11
	LINC00240-RP11-588K22.2	0.52	2.09e-05	0.17	0.55
	LINC00240-AC156455.1	0.61	2.95e-07	0.35	0.03
	AP000525.9-RP11-588K22.2	0.49	8.17e-05	0.14	0.55
	AP000525.9-AC156455.1	0.47	8.17e-05	0.19	0.49
	RP11-588K22.2-AC156455.1	0.76	1.78e-12	0.15	0.55

CC represents the Pearson correlation coefficient.

Then, 3,036 and 3,551 differentially expressed coding genes were respectively identified in the subtype 1 and subtype 2. The differentially expressed genes (DEGs) were divided into three groups, including the DEGs only in the subtype 1 (849 genes), the DEGs only in the subtype 2 (1,364 genes) and the DEGs in both subtypes (2,187 genes). Enrichment analysis on each group of DEGs was performed based on GO terms and KEGG pathways. The DEGs in both subtypes were more significantly enriched for cancer-related pathways (such as “Cell cycle”, “DNA replication” and “ECM-receptor interaction” etc.) than the other two groups (Figure 1C, 1D). Nevertheless, the DEGs in the subtype 1 were more significantly enriched for the function of response to wounding (“response to wounding” and “regulation of response to wounding”) than the other two groups (Figure 1C). Many studies have shown that biological process of response to wounding was closely related to ESCC [20, 21]. High-level expression of epidermal growth factor receptor (*EGFR*), as the main positive regulator involved in wound re-epithelialization, is associated with well-differentiated ESCC [21, 22]. Our study revealed that *EGFR* was up-regulated (fold-change = 2.14) in the subtype 1 of ESCC, but not obviously up-regulated (fold-change = 1.59) in the subtype 2, which indicated that *EGFR* might be a potential molecular marker to identify the subtypes of ESCC.

### Overall of ESCC subtype-specific differential mRNA-lncRNA crosstalk networks

**Figure 2:**
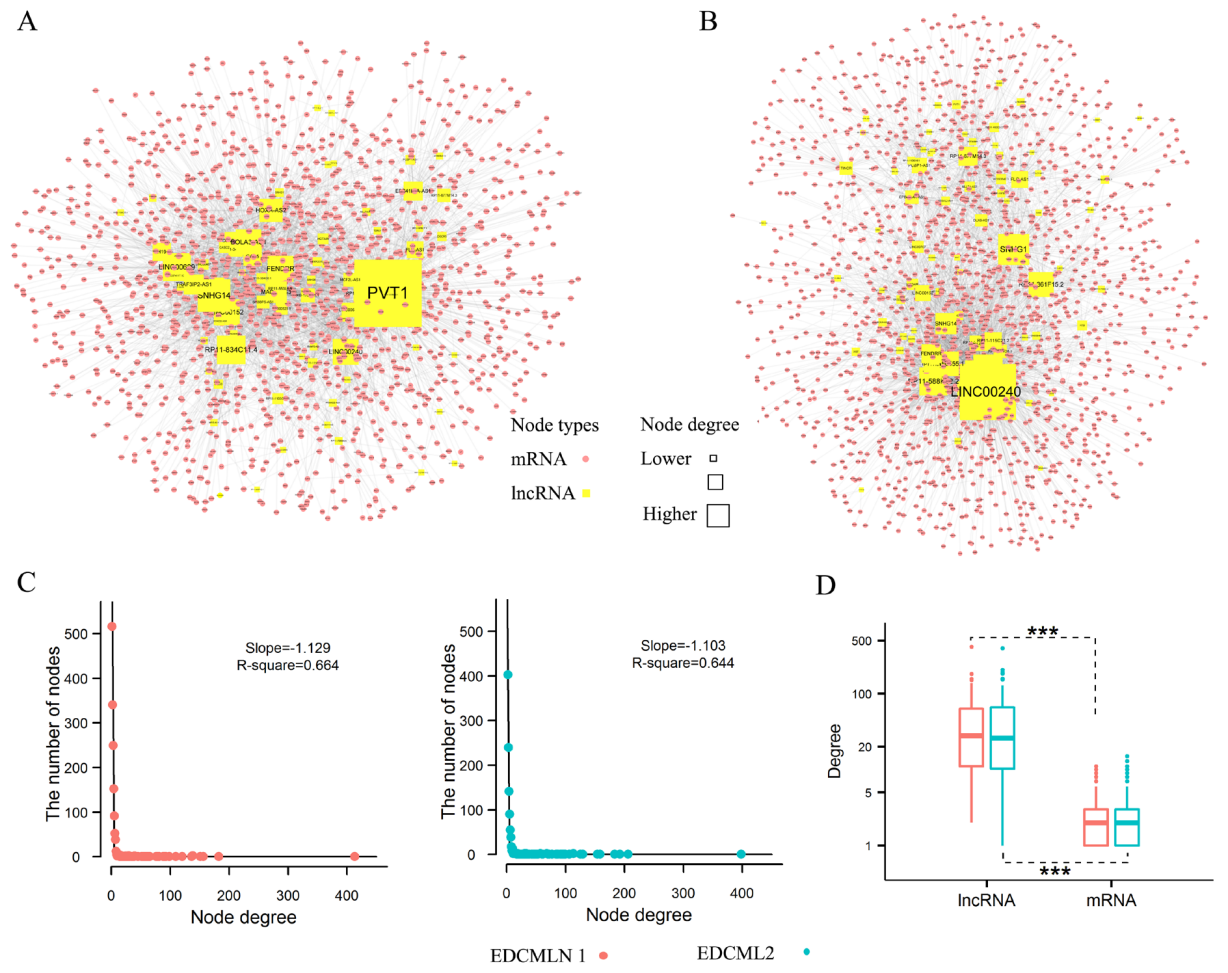
ESCC subtype-specific differential mRNA-lncRNA crosstalk networks based on ceRNA hypothesis and their properties **A**. Global view of the differential mRNA-lncRNA crosstalk network 1, which consists of 1,524 nodes and 3,699 links. **B**. Global view of the differential mRNA-lncRNA crosstalk network 2, which consists of 1,774 nodes and 4,131 links. **C**. Degree distribution of two differential mRNA-lncRNA crosstalk networks. **D**. The difference between degree of lncRNAs and mRNAs nodes. Wilcoxon test assessed the different significance.

Based on ceRNA hypothesis, we respectively constructed ESCC subtype-specific differential mRNA-lncRNA crosstalk networks. The networks were constructed by integrating prior knowledge of miRNA-lncRNA interactions [14], miRNA-mRNA interactions [14, 23] and expression profiles, and searching the most significant changes of mRNA-lncRNA crosstalks between ESCC and adjacent normal tissues. There were 1,449 coding genes, 75 lncRNA genes and 3,699 edges (4,786 mRNA-lncRNA differentially co-expressed links (DCLs)) in the network 1 of subtype 1 (Figure 2A), and 1,688 coding genes, 86 lncRNA genes and 4,132 edges (4,868 mRNA-lncRNA DCLs) in the network 2 of subtype 2 (Figure 2B). As observed, the change of crosstalks between mRNAs and lncRNAs formed a scale-free structure typical of transcriptional regulatory networks (Figure 2C). In the two networks, degree of lncRNAs were significantly more than coding genes (*p* < 2.2e-16, Wilcoxon test) (Figure 2D). Moreover, crosstalks between mRNAs and lncRNAs tended to disappear rather than appear in ESCC. 77.1% and 83% DCLs were separately found as the “loss” of crosstalks in the subtype 1 and subtype 2, whereas, only 20.9% and 14.7% DCLs were identified as the “gain” of crosstalks in the two subtypes (Table S3).

In addition, compared to high proportion of overlapping coding genes and lncRNAs, few overlapping edges (mRNA-lncRNA DCLs) in the two networks (Table S3) were found. Further analysis revealed that the difference of crosstalks between mRNAs and lncRNAs also existed in adjacent normal tissues of the two subtypes of ESCC (Table S4). Recent study showed that histologically-normal cancer-adjacent tissue, which influences recurrence risk, could reflect the intrinsic tumor subtypes of breast cancer [24]. So the difference of mRNA-lncRNA crosstalks in adjacent normal tissues might result in different local recurrence rates and prognosis after surgery of two subtypes of ESCC.

### The hub nodes of differential mRNA-lncRNA crosstalk networks

Identification of important nodes of differential mRNA-lncRNA crosstalk networks was based on four topological properties (detail in Methods). lncRNAs almost occupied the top 10 of each property and the top-ranked lncRNAs had striking difference between the two subtypes (Table 1). Several cancer-associated lncRNAs were found, which have rarely been reported in ESCC (Table 1). Except that *H19* could promote ESCC cell proliferation and metastasis [25], *PVT1* as a rising star among oncogenic lncRNAs has been reported in plenty of cancers [26–28]. *LINC00152* could directly bind to *EGFR* and activate *EGFR* signaling pathway [29] and *FENDER* could suppress cell invasion and migration by downregulating *FN1* and *MMP2/MMP9* expression in gastric cancer [30]. *HOXA-AS2* acts as an apoptosis repressor in promyelocytic leukemia cells [31], and *SNHG1* has also been suggested as a new biomarker for lung cancer and prostate cancer [32]. Moreover, cancer-associated lncRNAs (collected by Lnc2Cancer [33]) were mapped to the two networks. We found cancer-associated nodes had significantly higher degrees, betweenness and closeness centrality than other nodes in the network 1 (Figure S1). Thus, all these lncRNAs as the hub nodes of the differential mRNA-lncRNA crosstalk networks may be essential for ESCC development and progression.

To uncover the hub miRNAs likely involved in the two networks, miRNAs were ranked according to their frequency of mediating mRNA-lncRNA crosstalks. 90% of the top 10 miRNAs were disease-associated miRNAs (collected by miR2Disease [34]) (Table S5). Surprisingly, we found well-known *miR-15a/16-1* cluster appeared in the both subtypes. *MiR-15a/16-1* cluster within 0.5 kb at chromosome position 13q14 target several oncogenes (including *BCL2*, *CCNE1* and *CCNB1,* which also existed in the two networks), to suppress cell cycle progression and proliferation in several malignant tumors [35–37]. In addition, we found 2 members (*miR-17-5p*, *miR-20a*) of *miR-17-92* cluster only appeared in the subtype 1. *MiR-17-92* cluster which locates on the *C13orf25* gene is overexpressed in ESCC and usually promote cancer cell proliferation [38]. Then we validated the expression of these two clusters in an independent data set (detail in Methods), and found *miR-17-5p*, *miR-20a* and *miR-16-5p* were upregulated in ESCC. So, dysregulated *miR-17-92* and *miR-15a/16-1* clusters might result in the change of crosstalks between mRNAs and lncRNAs in the pathogenesis of ESCC.

### Dysregulation of lncRNAs as ceRNAs in the two subtypes

**Figure 3:**
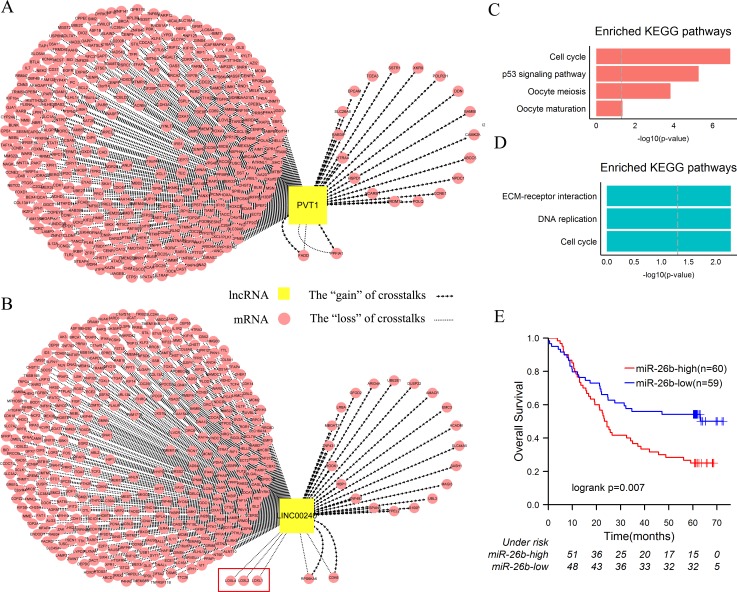
Dysregulation of PVT1 and LINC00240 as ceRNAs in ESCC **A**. The sub-network of network 1, including *PVT1* and its first neighbor mRNAs*.* **B**. The sub-network of network 2, including *LINC00240* and its first neighbor mRNAs. **C**. KEGG enrichment analysis of the first neighbor mRNAs of *PVT1*. **D**. KEGG enrichment analysis of the first neighbor mRNAs of *LINC00240.* **E**. In the independent dataset, patients were divided into two groups according to median expression level of *miR-26b* (high *miR-26b* expression and low *miR-26b* expression). Kaplan-Meier survival analysis of two groups of patients shows the significantly different clinical outcomes. P values were determined by the log rank test.

In order to explicitly explore dysregulation of lncRNAs as ceRNAs involved in the two subtypes of ESCC, we selected *PVT1* and *LINC00240—*the foremost lncRNAs of two subtypes according to topological properties for research (Table 1). Enrichment analysis was firstly performed based on their first neighbor mRNAs in the two networks (Figure 3A, 3B). Two gene sets were significantly enriched for the function of cell cycle and cancer-associated pathways such as “P53 signaling pathway”, “Cell cycle” and “Pathway in cancer” etc. (Figure 3C, 3D, Figure S2). Moreover, 95.2% and 94.5% genes of the two gene sets with *PVT1* and *LINC00240* occurred as the “loss” of crosstalks respectively (Figure 3A, 3B).

Then, we ranked all the miRNAs according to their frequency of mediating the “loss” of crosstalks. In the subtype 1 of ESCC, 38.2% “loss” of *PVT1*-associated crosstalks were likely mediated by *miR-186-5p* (Figure S3). It has been suggested that *miR-186* could inhibit the cell proliferation, migration and invasion of non-small cell lung cancer by modulating pituitary tumor-transforming gene-1 (*PTTG1*) [39]. In ESCC, *miR-186* and *PTTG1* that function as tumor suppressor and oncogene always are downregulated and upregulated respectively [40, 41]. Meanwhile, *miR-186*-*PVT1* interaction has been verified in several cell lines [14, 42], which implied that *PVT1* function as *miR-186* sponge and crosstalks between *PVT1* and *miRNA-186* target genes exist in various biological processes. In our study, although *PVT1* and *PTTG1* were both overexpressed in ESCC, positive correlation between them disappeared during the development of ESCC (Figure S4A, S4C). Therefore, we speculated that *miRNA-186*-mediated crosstalk between *PVT1* and *PTTG1* existed in adjacent tissues and maintained normal functions, and the “loss” of *PVT1*-*PTTG1* crosstalk might act as a driver event implicated in development of the subtype 1 of ESCC. Similar results—the “loss” of *miR-200* family mediated crosstalks between *PVT1*-mRNAs have also been proposed in breast cancer [43].

**Figure 4:**
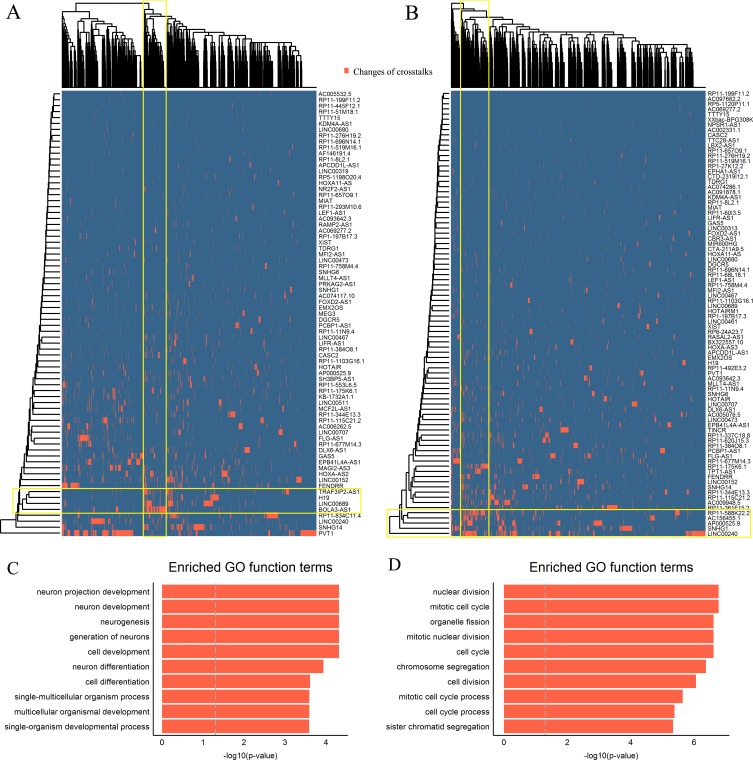
ESCC subtype-specific modules of differential mRNA-lncRNA crosstalks **A**.-**B**. The bidirectional hierarchical heat maps of two differential mRNA-lncRNA crosstalk networks. The overlaps of yellow rectangles represent functional modules. **C**.-**D**. Top 10 significantly enriched GO terms of two modules.

In the subtype 2 of ESCC, 49.2% “loss” of *LINC00240*-associated crosstalks were possibly mediated by *miR-26b-5p* (Figure S3). Downregulation of *miR-26b-5p* could inhibit proliferation and induce cell-cycle arrest in ESCC [44]. In the independent microarray dataset, we found that expression of *miR-26b-5p* in well-differentiated cancer cells was significantly lower than poorly differentiated (*p* = 0.004, *t*-test). Furthermore, the patients were divided into two groups according to median expression level of *miR-26b-5p* and survival analysis was performed on the two groups. Patients with the higher expression level of *miR-26b-5p* had significantly shorter overall survival rate than those with the lower expression level (5-year survival rate: 25.0% *vs*. 52.0%, *p* = 0.008) (Figure 3E). Meanwhile, in the subtype 2, the “loss” of *miR-26b-5p*-mediated LINC00240-associated crosstalks accompanied the bad prognosis. So we proposed a bold hypothesis that the increased *LINC00240* are not necessarily binding to *miR-26b-5p* and accordingly overexpressed *miR-26b-5p* as an oncogene result in poor clinical outcome of the subtype 2. In order to verify this assumption, we compared the expression levels of target genes of *miR-26b-5p* in the two subtypes of ESCC samples. Kruppel-Like Factor 3 (Basic) (*KLF3*), which is an important transcriptional repressor involved in cell growth, apoptosis, and angiogenesis [45, 46], was significantly downregulated in the subtype 2 (fold-change = 0.7, q-value = 4.3e-4). Previous study suggested that low expression of *KLF3* was associated with bad prognosis of uterine cervical cancer [46]. Therefore, the “loss” of *miR-26b-5p*-mediated *LINC00240-KLF3* crosstalk was probably implicated in tumorigenesis of the subtype 2 of ESCC.

When comparing enriched pathways based on neighbor mRNAs of *PVT1* and *LINC00240*, extracellular matrix (ECM)-receptor interaction was the most significantly enriched pathway in the subtype 2 (Figure 3C, 3D). Upregulated *LOX*-mediated ECM remodeling is a poor prognostic marker in breast cancer and head and neck cancer [47, 48]. In ESCC, high *LOXL2* expression is a poor prognostic marker and high level of *LOXL4* is closely correlated with the poor differentiation [49]. In present study, the “loss” of crosstalks between *LINC00240* and 3 members of *LOX* gene family (including *LOXL1*, *LOXL2* and *LOXL4*) were found in the subtype 2 of ESCC (Figure 3B). Experimental data have shown that *LINC00240* and *LOXL2* possibly compete for *miR-26b-5p* binding [14], and overexpression of *miR-26b-5p* served as a poor prognostic marker in the subtype 2 of ESCC. Likewise, *LINC00240* and *LOXL1* or *LOXL4* possibly compete for *miR-124-3p* binding [14], which also has a significant difference in degree of differentiation of ESCC [50]. Thus, in the subtype 2, the “loss” of miRNA-mediated crosstalks between *LINC00240* and *LOX* gene family might result from *LOX-*mediated dysregulation of ECM.

### Module analysis of lncRNAs as ceRNAs in the two classes

To further investigate the cooperative function of multiple lncRNAs as miRNAs sponges, bidirectional hierarchical clustering on the two networks was respectively performed. We found two DCL modules in the heat map of the two subtypes. The first module of the subtype 1 contained 4 lncRNAs (*LINC00689, BOLA3-AS1, H19 and TRAF3IP2-AS1*) and 126 mRNAs (Figure 4A), and the second module of the subtype 2 contained 5 lncRNAs (*LINC00240, SNHG1, AP000525.9, RP11-588K22.2, AC156455.1*) and 172 mRNAs (Figure 4B).

The coding genes in the first module were significantly enriched for GO terms related to neuron development (Figure 4C). We ranked these coding genes according to the degree in the first module and found 6 hub coding genes (Table S6). Among them, neuronal growth regulator 1 (*NEGR1*) and basic helix-loop-helix family member e22 (*BHLHE22*) are crucial transcription factors associated with neuron development, which as tumor suppressors were downregulated in the subtype 1 of ESCC [51, 52]. Among 4 lncRNAs, *LINC00689, BOLA3-AS1* and *TRAF3IP2-AS1*, were as the most frequent combination in crosstalks (Table S6). Any two lncRNAs within them were significantly positively correlated in adjacent tissues, whereas lost their correlations in tumor tissues (Table 2).

In the second module, the coding genes were significantly enriched for GO terms related to mitotic cell cycle (Figure 4D). Similarly, we found 11 hub coding genes (Table S6), including apoptosis-regulatory gene (*BID)* and DNA double-strand break repair gene (*RAD51AP1)* etc., which are closely related to cancer development [53, 54]. The most frequent lncRNA combination of module 2 including *LINC00240*, *AP000525.9*, *RP11-588K22.2* and *AC156455.1* also lost their correlations during the development of ESCC (Table 2). Moreover, the “loss” of crosstalks between the hub coding genes and lncRNAs were found in both modules as well. These together suggested that collaborative crosstalks between these lncRNAs and the key coding genes within the functional modules existed in normal tissue and the “loss” of them might participate in ESCC development.

## DISCUSSION

The ceRNA hypothesis suggests that miRNA-mediated crosstalks between mRNAs and lncRNAs are well-organized. However, molecular classification of ESCC based on mRNA-lncRNA expression data has never been reported. Our study identified two biologically and clinically relevant subtypes of ESCC based on expression profiles of lncRNA and mRNA. The functional characteristic of subtypes 1 was closely associated with “response to wounding”. *EGFR* is a crucial regulator in wound healing and tumor growth, and overexpressed *EGFR* was related to the process of ESCC infiltration in the early stages of carcinogenesis [55]. In present study, the hub lncRNA—*LINC00152* was upregulated in the subtype 1 of ESCC as well as *EGFR*. *LINC00152* can directly bind to *EGFR*, resulting in activation of *PI3K/AKT* signaling pathway in gastric cancer [29]. Intriguingly, the “gain” of miRNA-mediated crosstalk between *LINC00152* and *EGFR* was also found in the subtype 1 of ESCC. It implied that these two genes might form a positive feedback loop to enhance *EGFR* downstream signaling pathway in tumor cell. Currently, anti-*EGFR* inhibitors have been evaluated in ESCC [56]. Thus, inhibition of activity of *LINC00152* may provide a new opportunity for therapeutic strategy.

Differential co-expression analysis (DCEA) is proposed to understand the roles of genes interconnection in complex human diseases as a complementary technique to the traditional differential expression analysis (DEA) [57]. Combination of DCEA and DEA, a great many “loss” of miRNA-mediated crosstalks between mRNAs and lncRNAs were detected during the tumorigenesis of ESCC. The most “loss” of crosstalks between *PVT1*-mRNAs in the subtype 1 of ESCC were mediated by *miR-186*, which was inconsistent with previous report in breast cancer [43]. One possible explanation for this difference is that experimentally supported instead of predicted miRNA-lncRNA interactions was used. Another potential reason is that tissue-specific genes express in different adjacent normal tissues. A recent review tried to account for this “loss” of crosstalks in cancer, including lncRNA isoforms losing the specific MREs and preferential expressing the lncRNA isoforms without specific miRNA binding site [58]. Moreover, a single crosstalk between mRNA and lncRNA is mediated by several miRNAs, and dysregulation of any miRNAs may disrupt the crosstalk. Although these reasons require further confirmation, our results still discovered a number of meaningful “loss” of crosstalks between mRNAs and lncRNAs, which may serve as driver events in ESCC development.

In addition, the cooperation of multiple lncRNAs deserves our attention. More accurate prediction of patient survival has been acquired through combining lncRNA signatures in ESCC [6]. lncRNA-lncRNA synergistic networks were also discussed [33]. Our findings suggested that ceRNA network may partially account for lncRNA-lncRNA synergistic effects. In summary, our study depicted mRNA-lncRNA molecular portraits of ESCC. Analysis of subtype-specific differential mRNA-lncRNA crosstalk networks provided multiple perspectives on roles of lncRNAs involved in ESCC, and facilitated research in personalized medicine and potential new therapeutic targets for ESCC.

## MATERIALS AND METHODS

### Gene expression profile

lncRNA and mRNA expression profiles of cancer and adjacent normal tissues form 119 ESCC patients, and clinical data were downloaded from the GEO database under accession number of GSE53624 [6]. miRNA expression profiles of ESCC were downloaded from the GEO database under accession number of GSE43732 [59].

### Probe re-annotation

We have re-annotated probes from Agilent-038314 CBC Homo sapiens lncRNA + mRNA microarray V2.0 platforms (https://www.ncbi.nlm.nih.gov/geo/query/acc.cgi?acc = GPL18109). Human protein-coding transcript sequences and long non-coding transcript sequences were downloaded from the NCBI Reference Sequence Database (Refseq) and GENCODE database. The microarray probes were re-annotated as follow:

(1) The probes named as “CB*” and “RNA*” were aligned to protein-coding transcript sequences and long non-coding transcript sequences by BLASTn tools respectively.

(2) The probes that only perfectly matched to one transcript or multiple transcripts from same gene were reserved.

(3) The average expression values of multiple probes sets that mapped to the same gene or transcript were calculated.

Then, 18,755 mRNAs and 7,171 lncRNAs were annotated. The average expression levels of mRNAs and lncRNAs were 10.25 and 6.78 (log 2 transformation, Figure S5).

**Figure 5:**
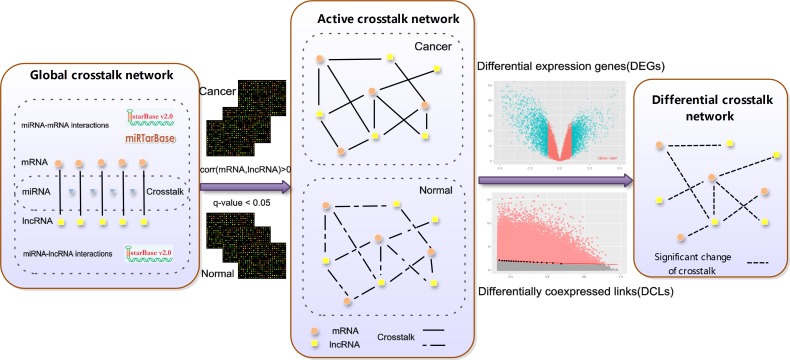
An integrative pipeline for construction of differential mRNA-lncRNA crosstalk networks based on ceRNA hypothesis Experimental-supported miRNA-lncRNA and miRNAs-mRNA interactions were downloaded from starBase and miRTarBase. miRNA-lncRNA and miRNA-mRNA pairs sharing the same miRNA form the global crosstalk network. If the Pearson correlation coefficient between competing lncRNA-mRNA pairs > 0 and q value < 0.05 in ESCC tumor or adjacent tissues, the competing lncRNA-mRNA pairs were retained and formed the active crosstalk network. Finally, subtype-specific differential mRNA-lncRNA crosstalk networks based on ceRNA hypothesis are respectively constructed by integrating DEGs and DCLs.

### Differentially expressed genes (DEGs)

Moderated paired *t*-test within the linear models of R package Limma was used to assess differential expression between tumor and adjacent normal tissues [60]. DEGs were identified if the absolute value of fold-change > 2 and adjust *p*-value < 0.05 (Benjamini-Hochberg method to control the false discovery). 3,170 and 765 differentially expressed mRNAs and lncRNAs were retained in the subtype 1 of ESCC, meanwhile 3,697 and 878 differentially expressed mRNAs and lncRNAs were retained in the subtype 2 (Figure S6).

### ESCC subtypes classification based on mRNA-lncRNA expression profile

Considering the influence of heterogeneity among different patients, relative expression levels between tumor and normal tissues were used. Semi-non-negative matrix factorization (semi-NMF) consensus clustering was used to identify intrinsic subtypes of ESCC based on the top 30% variable mRNAs and lncRNAs. The best number of clusters was selected based on dispersion coefficients of semi-NMF (range of cluster k values from 1 to $\sqrt{\text{sample size}}$, 30 times rerun of each cluster k and 200 iterations of each run). The value of the fitting residual of consensus clustering terminated at 1e-6 and consensus clustering iterated 1,000 times. All above were implemented by the NMF toolbox in MATLAB [61].

### Construction of differential mRNA-lncRNA crosstalk network based on ceRNA hypothesis

Global mRNA-lncRNA crosstalk network and active mRNA-lncRNA crosstalk network were built before constructing the differential mRNA-lncRNA crosstalk network (Figure 5). First 668,645 human experimentally validated miRNA-mRNA interactions (2,650 miRNAs and 15,353 mRNAs) were downloaded from the starbase V2.0 database [14] and miRTarBase database [23], and the CLIP-Seq experimentally supported 10,212 miRNA-lncRNA interactions (227 miRNAs and 1,127 lncRNAs) were gotten from the starbase V2.0 database. 1,812,628 lncRNA-mRNA pairs (445 lncRNAs and 13,805 mRNAs) competition for common miRNAs binding constituted the global mRNA-lncRNA crosstalk network.

The competing lncRNA-mRNA pairs were retained if corr (mRNA, lncRNA) > 0 and q-value < 0.05 in ESCC tumor or adjacent tissues, which formed the active mRNA-lncRNA crosstalk network (q-value was estimated from the p-value of Pearson correlation coefficients using the Benjamini-Hochberg method). Identifying the significant changes of crosstalks between mRNAs and lncRNAs during the development of ESCC was simplified into searching the differentially co-expressed links (DCLs) between ESCC tumor and adjacent tissues. The DCLs were identified by the limit fold change model (LFC) [62]. LFC defined a fraction δ of links with highest log fold change (y) between maximum co-expression (x), and links lying above the fitted curve y = a + (b/x) are considered as DCLs. The top 20% significant changes of crosstalks between mRNAs and lncRNAs were retained (δ = 0.2, δ setting was tested in supplementary). Then we filtered the DCLs in which lncRNAs and mRNAs were not DEGs. Finally, the differential mRNA-lncRNA crosstalk network was constituted by merging multiple DCLs into one edge if they connected same coding genes and lncRNAs.

In addition, the DCLs were divided into two groups. Competing lncRNA-mRNA pairs had higher positive correlation in adjacent tissues than tumor tissues, which were defined as the “loss” of crosstalks. On the contrary, they were regarded as the “gain” of crosstalks.

### Topological features selected

Four topological features were selected to assess importance of node in the differential mRNA-lncRNA crosstalk networks.

(1) *Degree*. The number of edges linking to the given node.

(2) *Betweenness*. The number of shortest paths between any two vertexes that pass through the given node.

(3) *Closeness*. The average length of paths from given node to all other reachable nodes.

(4) *DCLs enrichment.* The DCL edges linking to the given node in the differential crosstalk network enriched the edges linking to the given node in the active crosstalk network. *DCLs enrichment* reflected the importance of nodes in differential regulation network [63]. It was measured by hypergeometric test as:

$$P=1-\sum_{i=0}^{m-1}\frac{\left(\begin{array}{c} M\\ i \end{array}\right)\left(\begin{array}{c} N-M\\ n-i \end{array}\right)}{\left(\begin{array}{c} N\\ n \end{array}\right)}$$

where, N represented the total number of edges in the active crosstalk network, n represented the total number of DCL edges in the differential crosstalk network, M represented the number of edges linking to the given node in the active crosstalk network and m represented the number of DCL sedges linking to the given node in the differential crosstalk network.

*Degree, Betweenness and Closeness* were calculated by Cytoscape and *DCLs enrichment* was implemented by R.

### Survival and cox regression analysis

Kaplan-Meier survival analysis was performed for the patients after surgery and statistical significance was assessed by the log-rank test. All analysis was implemented by R using the survival package.

### Enrichment analysis

Gene Ontology and KEGG pathways enrichment analysis were implemented by R using annotation data packages (org.Hs.egGO, org.Hs.egPATH). Biology process terms and KEGG pathways with q-value < 0.05 were statistically significant (Benjamini-Hochberg method adjusted).

## SUPPLEMENTARY MATERIALS FIGURES AND TABLES


