# Supplementary material for: Construction of differential mRNA-lncRNA crosstalk networks based on ceRNA hypothesis uncover key roles of lncRNAs implicated in esophageal squamous cell carcinoma
Source: Oncotarget. 2016 Dec 9;7(52):85728–40. doi: 10.18632/oncotarget.13828 (PMC5349869; doi:10.18632/oncotarget.13828)
Supplement: Supplementary file 1 [file oncotarget-07-85728-s001.pdf]

Construction of differential mRNA-lncRNA crosstalk networks based on ceRNA hypothesis uncover key roles of lncRNAs implicated in esophageal squamous cell carcinoma

Supplementary Material

Assessment of different values of δ for searching the significant changes of crosstalks between mRNAs and lncRNAs

Different values of δ in the limit fold change (LFC) model used to identify DCLs have been discussed, and 0.1 was the recommended numerical value of δ [1]. In present study, in view of crosstalks between mRNAs and lncRNAs coming from prior-knowledge rather than random mRNA-lncRNA pairs, the percentage of significant changes of crosstalks between mRNAs and lncRNAs (δ) was relaxed appropriately. Moreover, in the next analysis, the changes of crosstalks between mRNAs and lncRNAs that were not differential expressed genes (DEGs) were also filtered. Thus, we retained a slightly high percentage of significant changes of crosstalks and set δ = 0.2 in our study. To verify the reliability of our results, we reset δ = 0.1 and reconstructed two differential mRNA-lncRNA crosstalk networks. The top 10 genes in betweenness, degree, closeness and DCLs enrichment of two networks were shown in table S7. In addition, comparing hub genes of networks constructed by different values of δ, the high proportion of overlaps were found (Figure S7).

Table

Supplementary Table S1. Correlations between subtypes of ESCC and clinical features

Clinical features	P-value
Tumor grade	3.55e-05
Tumor stage	0.15
Alcohol use	0.06
Sex	0.48
Tobacco use	0.69
T stage	0.26
N stage	0.97
Arrhythmia	0.19
Pneumonia	0.54
Anastomotic leak	1
Adjuvant therapy	0.45

Fisher's exact test was used.

Supplementary Table S2. Contingency table of tumor grade versus subtypes of ESCC

Subtypes\Tumor grade	Well (obs./exp.)	Moderately (obs./exp.)	Poorly (obs./exp.)
Subtype1	13/10.5	37/29	4/14.5
Subtype2	10/12.5	27/35	28/17.5

Supplementary Table S3. The detail information of two differential mRNA-lncRNA crosstalk networks

	Network 1	Network 2	Overlapping		
			Num	Percentage 1	Percentage 2
Coding genes	1,449	1,688	777	54%	46%
lncRNAs	75	86	52	69%	60%
Edges	3,698	4,131	294	8%	7%
DCLs	4,786	4,868	209	4%	4%
The “gain”	1,019	786			
The “loss”	3,767	4,082			

Supplementary Table S4. Overlaps of the “loss” crosstalks and positive correlations between mRNAs and lncRNAs in the adjacent tissues of two subtypes of ESCC

		The “loss” crosstalks between mRNAs and lncRNAs	
		Subtype 1	Subtype 2
Positive correlation between mRNAs and lncRNAs	Subtype 1	3,767	2,127
	Subtype 2	1,583	4,082

Supplementary Table S5. The top 10 miRNAs mediated differential mRNA-lncRNA crosstalk networks

Network 1			Network 2		
Names	Frequency	Percentage	Names	Frequency	Percentage
**miR-16-5p**	406	10.98%	**miR-26b-5p**	478	11.57%
**miR-17-5p**	362	9.79%	**miR-16-5p**	459	11.11%
**miR-93-5p**	359	9.71%	**miR-15b-5p**	407	9.85%
**miR-106a-5p**	339	9.16%	**miR-497-5p**	404	9.78%
**miR-15b-5p**	338	9.14%	**miR-424-5p**	388	9.39%
**miR-15a-5p**	335	9.06%	**miR-15a-5p**	386	9.34%
**miR-20a-5p**	335	9.06%	**miR-195-5p**	356	8.62%
**miR-20b-5p**	330	8.92%	**miR-26a-5p**	287	6.95%
**miR-424-5p**	330	8.92%	miR-1297	269	6.51%
**miR-106b-5p**	330	8.92%	miR-103a-3p	245	5.93%

Disease-related miRNAs were highlighted in bold font.

Supplementary Table S6. Hub coding genes and associated lncRNAs in two modules

	Hub coding genes	Degree	Associated-lncRNAs
Module 1	*NTNG1*	3	*BOLA3-AS1; TRAF3IP2-AS1;LINC00689*
	BHLHE22	3	*BOLA3-AS1; TRAF3IP2-AS1;LINC00689*
	UTRN	3	*BOLA3-AS1; TRAF3IP2-AS1;LINC00689*

	ANK2	3	*BOLA3-AS1; TRAF3IP2-AS1;LINC00689*
	EDNRB	3	*BOLA3-AS1; TRAF3IP2-AS1;LINC00689*
	NEGR1	3	*BOLA3-AS1; TRAF3IP2-AS1;H19*
Module 2	*GRB10*	4	*LINC00240; SNHG1; RP11-588K22.2; AC156455.1*
	LPCAT1	4	*LINC00240; SNHG1; RP11-588K22.2; AC156455.1*
	BID	4	*LINC00240;AP000525.9;RP11-588K22.2; AC156455.1*
	GREM2	4	*LINC00240; AP000525.9; RP11-588K22.2; AC156455.1*
	DYNC1I1	4	*LINC00240; AP000525.9; RP11-588K22.2; AC156455.1*
	DCBLD2	4	*LINC00240; AP000525.9; RP11-588K22.2; AC156455.1*
	HOXD8	4	*LINC00240; AP000525.9; RP11-588K22.2; AC156455.1*
	TMEM38B	4	*LINC00240; AP000525.9; RP11-588K22.2; AC156455.1*
	RAD51AP1	4	*LINC00240; AP000525.9; RP11-588K22.2; AC156455.1*
	PPT1	4	*LINC00240; AP000525.9; RP11-588K22.2; AC156455.1*
	DPY19L1	4	*LINC00240; SNHG1; AP000525.9;AC156455.1*

Supplementary Table S7. The top 10 genes in betweenness, degree, closeness and DCLs enrichment of two differential mRNA-lncRNA crosstalk networks using δ = 0.1

	Betweenness	Degree	Closeness	*DCLs* enrichment	Overlaps
Network 1	***PVT1***	***PVT1***	***PVT1***	***PVT1***	***PVT1***
	SNHG14	*LINC00689*	*SNHG14*	*LINC00689*	*SNHG14*
	LINC00689	*SNHG14*	***FENDRR***	*SNHG14*	*RP11-834C11.4*
	RP11-834C11.4	*RP11-834C11.4*	*RP11-834C11.4*	***HOXA-AS2***	*LINC00240*
	HOXA-AS2	***FENDRR***	*LINC00240*	***LINC00152***	***LINC00152***
	FENDRR	***HOXA-AS2***	*PPFIA1*	***FENDRR***	***FENDRR***
	LINC00240	***LINC00152***	*SLC16A14*	*RP11-834C11.4*	*LINC00689*
	LINC00152	*LINC00240*	*BOLA3-AS1*	***H19***	
	BOLA3-AS1	*MAGI2-AS3*	*LINC00689*	*RP11-115C21.2*	
	MAGI2-AS3	*BOLA3-AS1*	***LINC00152***	*LINC00240*	
Network 2	*LINC00240*	*LINC00240*	*LINC00240*	*LINC00240*	*LINC00240*
	SNHG1	*AP000525.9*	***SNHG1***	*AP000525.9*	***SNHG1***
	RP11-361F15.2	***SNHG1***	*AC156455.1*	*AC156455.1*	*AC156455.1*
	AC156455.1	*AC156455.1*	*PCDH19*	*RP11-677M14.3R*	*RP11-588K22.2*
	RP11-588K22.2	*RP11-588K22.2*	***FENDRR***	*RP11-361F15.2*	
	AP000525.9	*RP11-361F15.2*	*ZNF469*	*P11-588K22.2*	
	RP11-677M14.3	*AC009948.5*	*RP11-588K22.2*	*FLG-AS1*	
	FLG-AS1	*RP11-677M14.3*	*RFC3*	***SNHG1***	
	SNHG14	*SNHG14*	*GRB10*	*TINCR*	
	AC009948.5	***FENDRR***	*AC009948.5*	*RP11-175K6.1*	

Cancer-related lncRNAs were highlighted in bold font.

Figure S1.

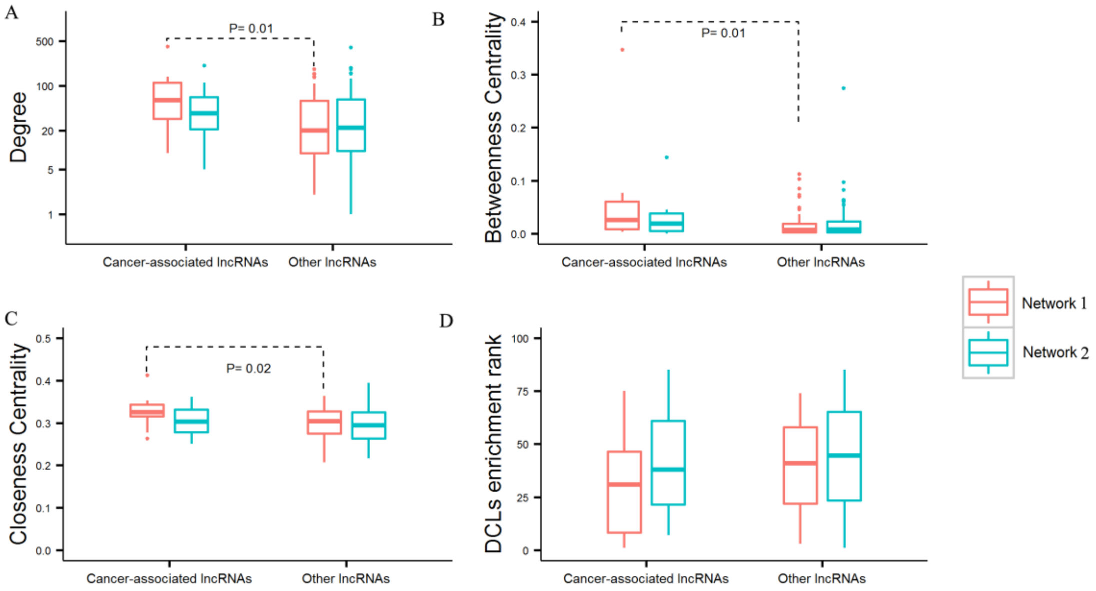

Figure S1. A-D. Difference between cancer-associated lncRNAs and other lncRNAs in four topological properties of differential mRNA-lncRNA crosstalk networks

Figure S2.

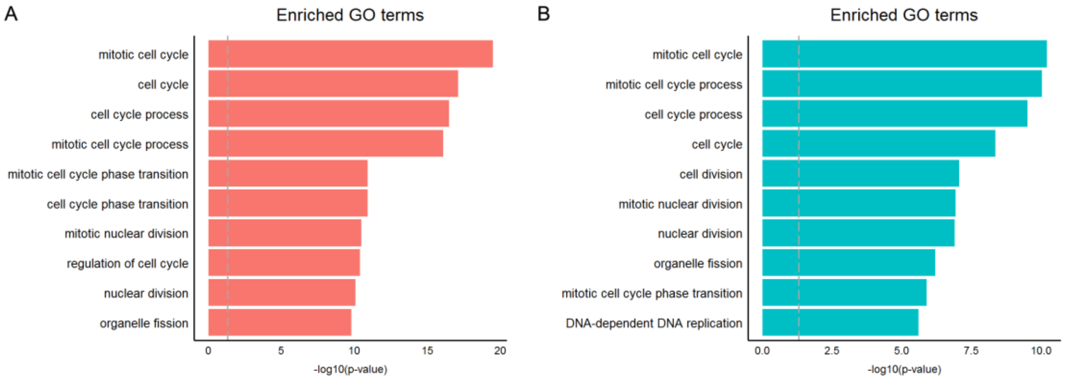

Figure S2. A-B. Top 10 significantly enriched GO terms based on their first neighbor mRNAs of *PVT1* and *LINC00240*.

Figure S3.

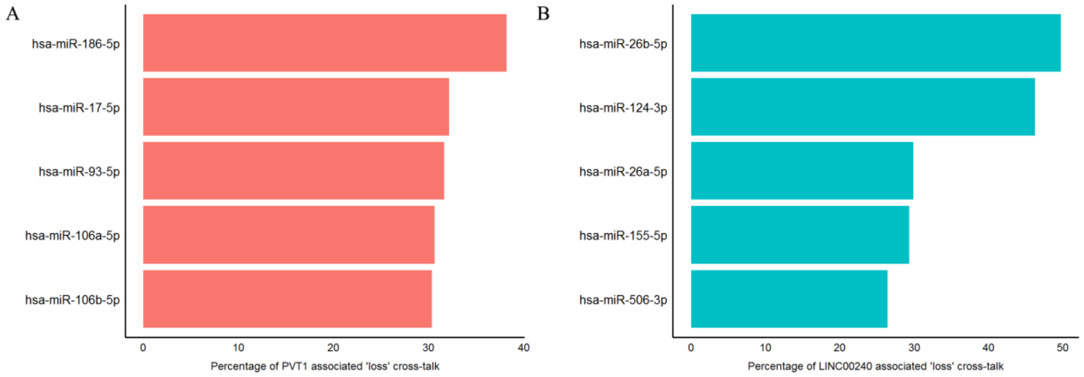

Figure S3. A-B. Top 5 miRNAs-mediated differential mRNA-lncRNA crosstalk networks.

Figure S4.

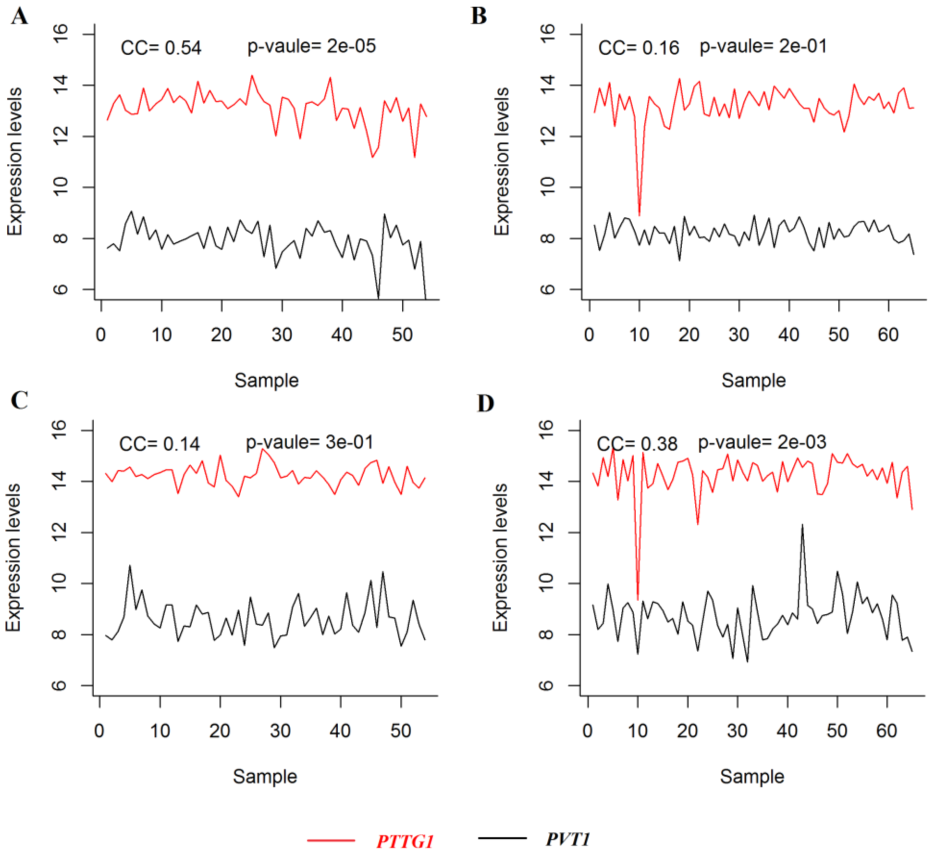

Figure S4. Correlations between *PTTG1* and *PVT1* in different tissues and subtypes of ESCC. A. Correlation between *PTTG1* and *PVT1* in adjacent tissues of the subtype 1 of ESCC. B. Correlation between *PTTG1* and *PVT1* in adjacent tissues of the subtype 2 of ESCC. C. Correlation between *PTTG1* and *PVT1* in the subtype 1 of ESCC. D. Correlation between *PTTG1* and *PVT1* in the subtype 2 of ESCC. CC represents the correlation coefficient. Pearson test assessed the different significance.

Figure S5.

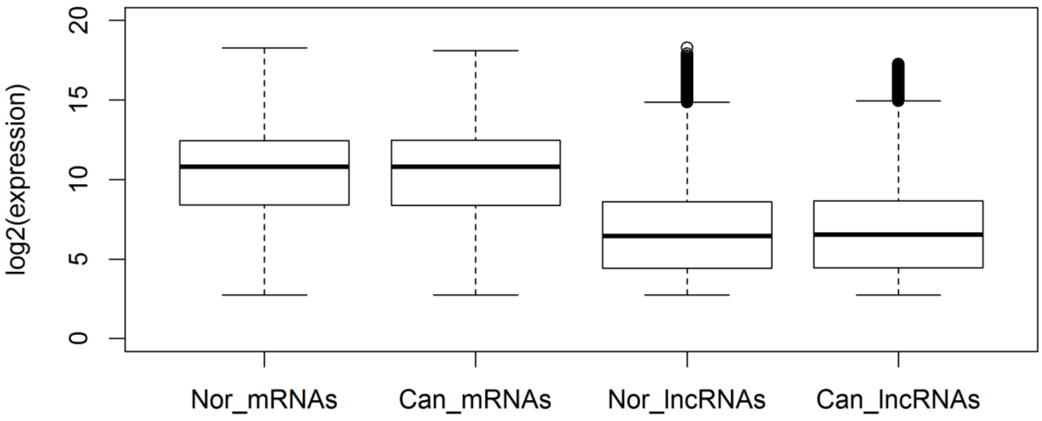

Figure S5. Expression levels of mRNAs and lncRNAs.

Figure S6

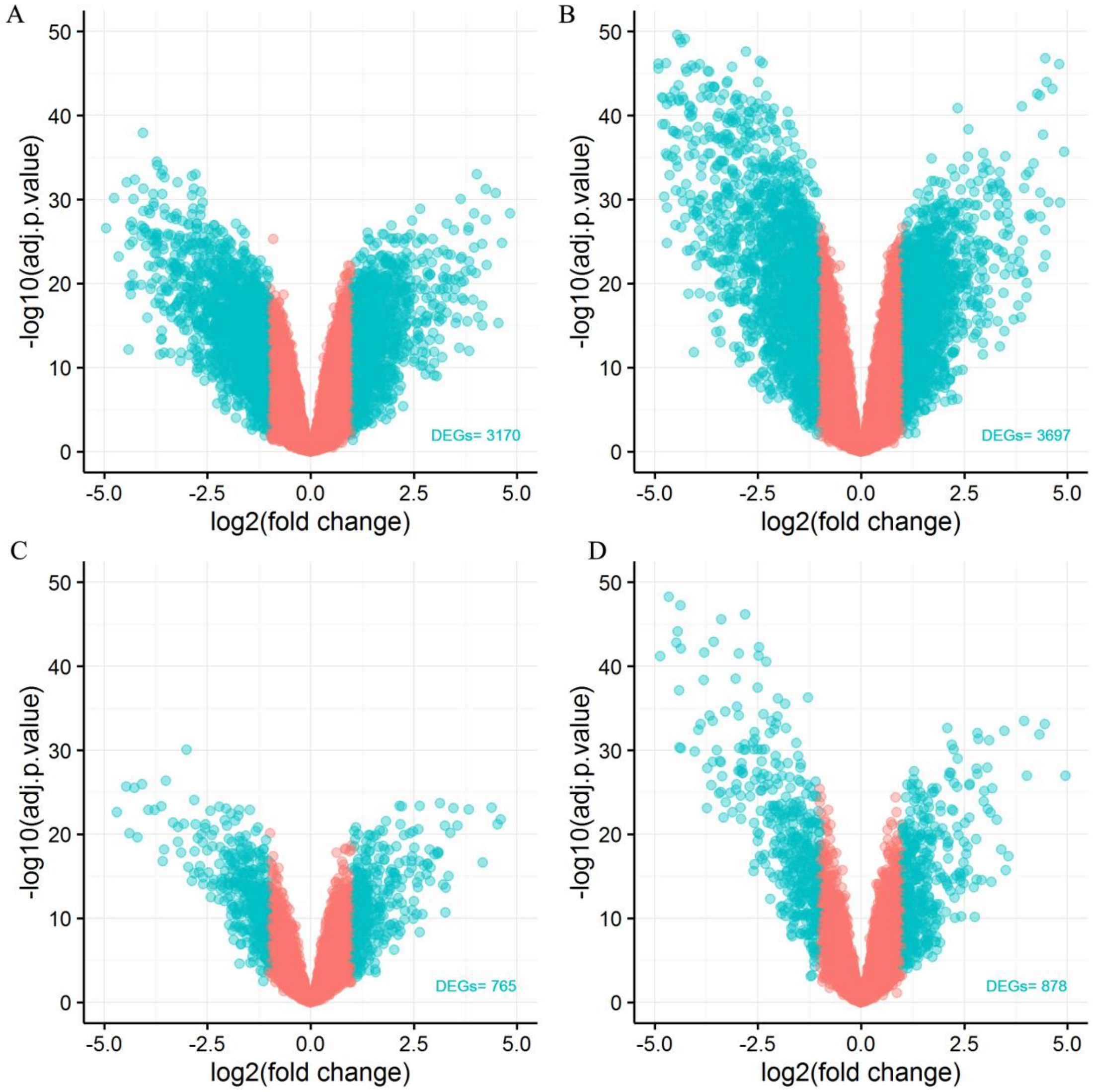

Figure S6. Differentially expressed genes (DEGs) in the two subtypes. A-B. Differentially expressed mRNAs in the subtype 1 and 2. C-D. Differentially expressed lncRNAs in the subtype 1 and 2.

Figure S7

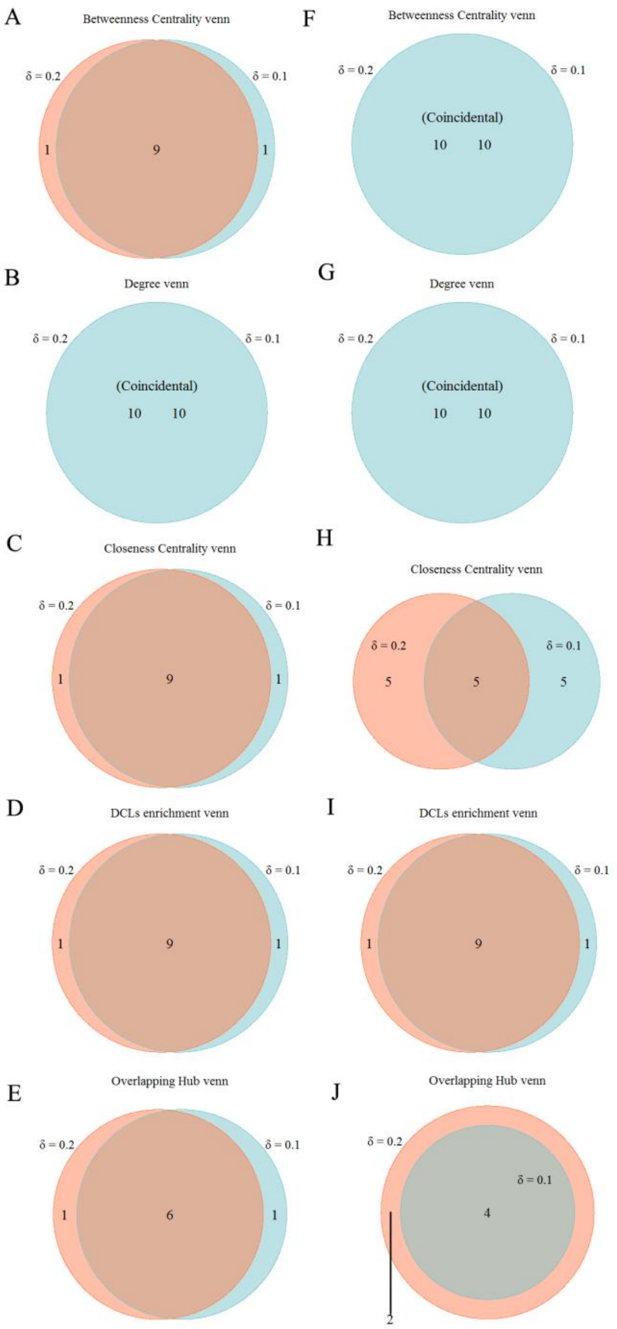

Figure S7. Consistency analysis of different values of δ**.** A-E. Venn diagram shows the overlaps of top 10 hub genes in different topological properties (betweenness, degree, Closeness, DCLs enrichment and overlapping hub) using different δ (0.1 and 0.2) to construct the differential mRNA-lncRNA crosstalk networks in the subtype 1 of ESCC. F-J. Venn diagram shows the overlaps of top 10 hub genes in different topological properties (betweenness, degree, Closeness, DCLs enrichment and overlapping hub) using different δ (0.1 and 0.2) to construct the differential mRNA-lncRNA crosstalk networks in the subtype 2 of ESCC.

REFERENCES

1. Yu H, Liu B-H, Ye Z-Q, Li C, Li Y-X and Li Y-Y. Link-based quantitative methods to identify differentially coexpressed genes and gene pairs. BMC bioinformatics. 2011; 12(1):1.
